# Moderate and strong static magnetic fields directly affect EGFR kinase domain orientation to inhibit cancer cell proliferation

**DOI:** 10.18632/oncotarget.9479

**Published:** 2016-05-19

**Authors:** Lei Zhang, Jihao Wang, HongLei Wang, Wenchao Wang, Zhiyuan Li, Juanjuan Liu, Xingxing Yang, Xinmiao Ji, Yan Luo, Chen Hu, Yubin Hou, Qianqian He, Jun Fang, Junfeng Wang, Qingsong Liu, Guohui Li, Qingyou Lu, Xin Zhang

**Affiliations:** ^1^ High Magnetic Field Laboratory, Chinese Academy of Sciences and University of Science and Technology of China, Hefei, Anhui 230026, China; ^2^ Hefei National Laboratory for Physical Sciences at The Microscale, University of Science and Technology of China, Hefei, Anhui 230026, China; ^3^ Laboratory of Molecular Modeling and Design, State Key Laboratory of Molecular Reaction Dynamics, Dalian Institute of Chemical Physics, Chinese Academy of Sciences, Dalian, Liaoning 116023, China; ^4^ Collaborative Innovation Center of Advanced Microstructure, Nanjing University, Nanjing, Jiangsu 210093, China

**Keywords:** EGFR, magnetic field, STM, cancer

## Abstract

Static magnetic fields (SMFs) can affect cell proliferation in a cell-type and intensity-dependent way but the mechanism remains unclear. At the same time, although the diamagnetic anisotropy of proteins has been proposed decades ago, the behavior of isolated proteins in magnetic fields has not been directly observed. Here we show that SMFs can affect isolated proteins at the single molecular level in an intensity-dependent manner. We found that Epidermal Growth Factor Receptor (EGFR), a protein that is overexpressed and highly activated in multiple cancers, can be directly inhibited by SMFs. Using Liquid-phase Scanning Tunneling Microscopy (STM) to examine pure EGFR kinase domain proteins at the single molecule level in solution, we observed orientation changes of these proteins in response to SMFs. This may interrupt inter-molecular interactions between EGFR monomers, which are critical for their activation. In molecular dynamics (MD) simulations, 1-9T SMFs caused increased probability of EGFR in parallel with the magnetic field direction in an intensity-dependent manner. A superconducting ultrastrong 9T magnet reduced proliferation of CHO-EGFR cells (Chinese Hamster Ovary cells with EGFR overexpression) and EGFR-expressing cancer cell lines by ~35%, but minimally affected CHO cells. We predict that similar effects of magnetic fields can also be applied to some other proteins such as ion channels. Our paper will help clarify some dilemmas in this field and encourage further investigations in order to achieve a better understanding of the biological effects of SMFs.

## INTRODUCTION

How magnetic fields influence biological systems is a fundamental question with potentially important relevance to environmental and medical exposure. Magnetic fields can be categorized into static magnetic field (SMF) and dynamic magnetic field (DMF), depending on whether the intensity and direction of magnetic field changes over time. According to their magnetic field strength, they are usually classified as weak, moderate and strong magnetic fields, although the exact categories vary in different research fields. For example, some define magnetic field of ≥ 1T to be strong SMF but some consider ≥ 3T to be strong SMF. Nevertheless, the geomagnetic (~0.05 mT) and the core part of most clinical Magnetic Resonance Imaging (MRI, ~0.2–7T) fields are SMFs with potential biological and medical importance.

Many studies have investigated biological effects of magnetic fields, with results that depend on multiple factors including field frequency, intensity, exposure time, and dynamics. SMFs have been shown to inhibit cancer cell proliferation in multiple studies, but the mechanism was unclear [1–7]. Cancer cells often proliferate in response to signaling from Receptor Tyrosine Kinases (RTKs), and the effect of magnetic fields on EGFR phosphorylation was the subject of several studies [8–10]. A 0.4 mT 50 Hz and a 2 μT 1.8GHz pulsed magnetic fields (electromagnetic waves frequency) both increased EGFR phosphorylation, which were reversed by incoherent (“noise”) magnetic fields of the same intensities [9, 10]. These studies demonstrate that EGFR is one of the targets for magnetic field. However, the molecular mechanisms underlying these magnetic field-induced EGFR activity changes were not explored.

EGFR is a membrane receptor protein whose orientation is tightly controlled and correlated with its activity. It is an oncoprotein, as are multiple proteins in the EGFR signaling pathway, and several EGFR signaling inhibitors have been approved for cancer treatment. Recent structural and genetic studies revealed variants and details of EGFR activation mechanism [11–15]. An asymmetric inter-molecular interaction between two kinase domains is required for EGFR activation [11]. Additionally, EGFR activation also requires interactions between the transmembrane helices and juxtamembrane segments, which release the inhibition by the plasma membrane [14, 15]. More interestingly, different extracellular ligands can trigger differential orientation changes in the juxtamembrane regions of EGFR, which lead to diverse downstream activities [12, 13]. Therefore, the EGFR activation process is exceptionally sensitive to orientation. Even a small change in its domain orientation could affect the intermolecular interaction between EGFR kinase domains, transmembrane helices and juxtamembrane regions, which subsequently affects its activation and functions.

SMFs are able to align large biological samples that have diamagnetic anisotropy, such as microtubule polymers, collagen and nucleic acid chains [16–29]. Computer-based Molecular Dynamics (MD) analysis shows that polyethylene chains of only a few hundred atoms, can be strongly aligned and stretched to a straight line by a 25T SMF within 10 ps [30], which demonstrates that the reorientation effect of SMF can be strong and significant. For proteins, the diamagnetic anisotropy is largely due to the alpha helix, beta sheet, aromatic rings and even peptide bonds [25, 26, 31]. A single peptide bond has weak diamagnetic anisotropy [25] but when they link together in a fixed and organized orientation in alpha helix or beta sheet, the overall diamagnetic anisotropy can be much stronger [26]. Although most proteins have very weak diamagnetic anisotropy, their response to the magnetic field can be amplified when they are constrained in membrane sheets, in liquid crystal phase, or in ordered polymers where the additive diamagnetic anisotropy can be significant. In addition, proteins that have a large number of aromatic amino acids (phenylalanine, tyrosine and tryptophan) are expected to have larger diamagnetic anisotropy because the aromatic rings have larger diamagnetic anisotropy than regular peptide bond.

Since EGFR is a membrane located RTK whose activity is very sensitive to subtle orientation changes, we suspected that it could be affected by static magnetic field. Its activity has been shown to be affected by different dynamic magnetic fields [8–10] and here we used static magnetic field, which is a more reliable system to investigate the underlying molecular mechanism. We found that EGFR autophosphorylation is directly inhibited by SMFs *in vitro*. Transfecting wild-type (wt), but not kinase-dead EGFR into CHO (Chinese hamster ovary) cells renders them sensitive to SMFs. This indicates that EGFR can be one of the key factors for cellular responses to SMF, especially for EGFR-expressing cancer cells. Scanning tunneling microscopy (STM) and molecular dynamics (MD) simulation show that SMFs can change the orientation of EGFR kinase domain proteins, which prevents them from forming asymmetrical dimers required for activation. In addition, stronger intensity SMFs (3-9T) have more obvious effects than moderate intensity SMFs (1T).

## RESULTS

### Static magnetic fields inhibit EGFR kinase activity to inhibit cell proliferation

**Figure 1:**
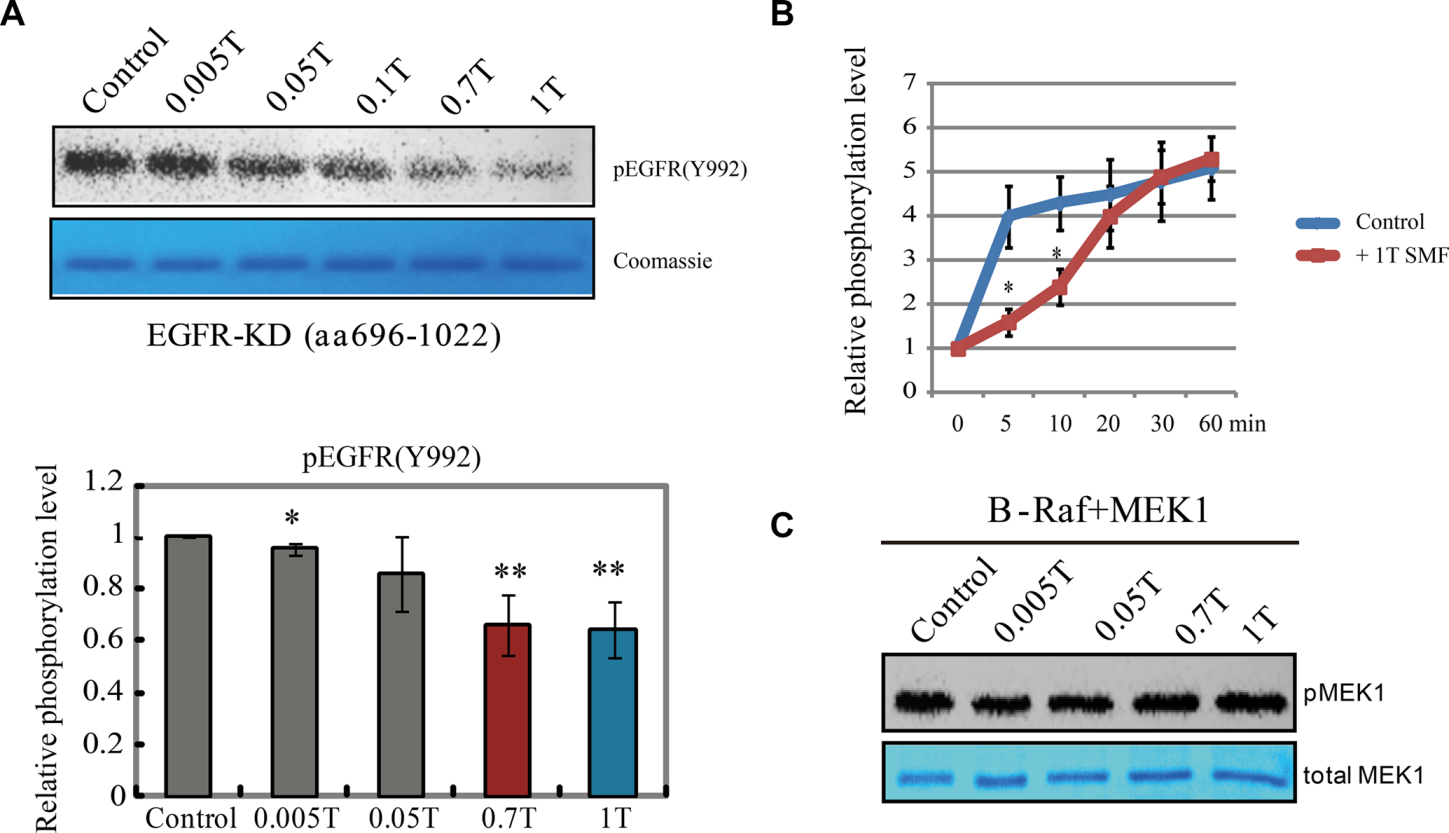
EGFR kinase activity is inhibited by moderate intensity static magnetic fields (SMFs) (**A**) *In vitro* kinase assays of EGFR kinase domain with different intensity SMFs. Representative Western blot using pEGFR (Y992) (top) and coomassie stain of total proteins (bottom) are shown. Quantifications of pEGFR (Y992) are shown in the bottom (*n* = 3). Error bars represent SD. *p < 0.05, **p < 0.01. (**B**) Time-course *in vitro* kinase assays of EGFR kinase domain with or without 1T SMF. Quantifications of the phosphorylation levels of pEGFR (Y992) are shown (*n* = 3). Error bars represent SD. *p < 0.05. (**C**) *In vitro* kinase assays of B-Raf kinase domain and its substrate MEK1 in the absence or presence of different intensity SMFs. Representative Western blot using pMEK1 (top) antibody and coomassie stain of total MEK1 protein (bottom) are shown. The control assays were carried out in the static magnetic field of the earth. Error bars represent SD. *p < 0.05, **p < 0.01.

We used the baculovirus system to express and purify the human EGFR kinase domain with a C-terminal tail (aa 696-1022) and an *in vitro* kinase assay to verify its activity (Figure S1A–S1C). Spontaneous ligand-independent EGFR autophosphorylation on tyrosine residues was inhibited by the EGFR specific inhibitor Pelitinib, which confirmed its enzyme activity (Figure S1D). We used a graded series of permanent magnets (0.005 to 1T) placed inside 37^°^C cell incubators to examine their influence on purified EGFR kinase activity. We found that its kinase activity was effectively inhibited by SMFs of 0.7T and 1T (Figure 1A). Time course experiments revealed a reduction in autophosphorylation rate, but not final extent (Figure 1B), suggesting the magnetic field affected the dynamics of the reaction. This is the first time a magnetic field was shown to directly inhibit the activity of isolated EGFR. In contrast, the phosphorylation of B-Raf, a member of the RAF family of serine/threonine protein kinases, on its substrate MEK1, was not affected by SMFs (Figure 1C).

**Figure 2:**
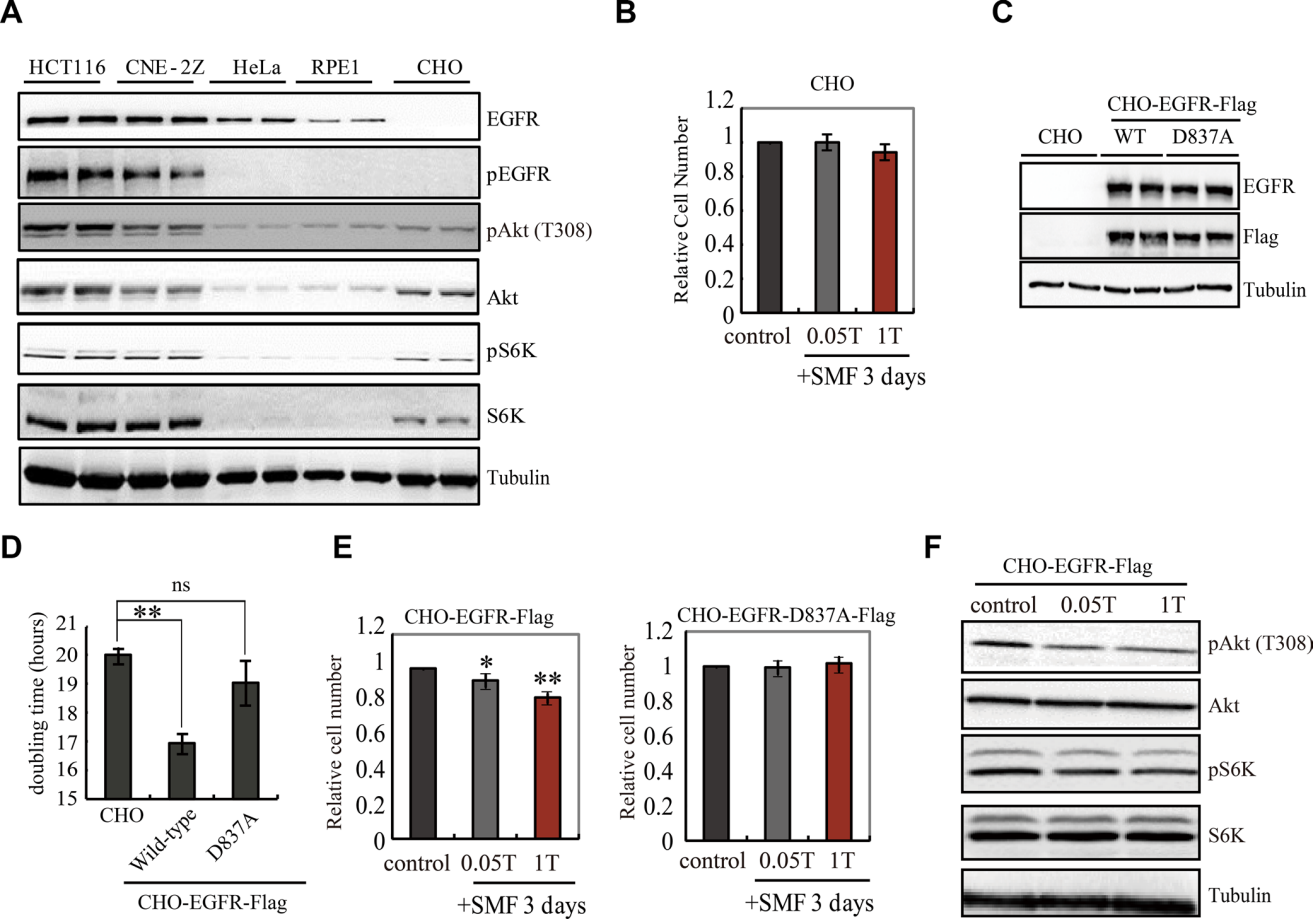
EGFR activity is important for SMF-induced cell growth inhibition (**A**) Representative Western blots are shown to compare the level of EGFR and pEGFR in five different cell lines. Samples were loaded in duplicate. (**B**) 0.05T and 1T SMFs do not affect CHO cells. Relative cell numbers of CHO cells after 3 days treatment in 0, 0.05, or 1T SMFs are shown. (**C**) Representative Western blots comparing CHO cells and CHO cells stably expressing wild-type EGFR (CHO-EGFR-Flag) or kinase-dead EGFR (CHO-EGFR-D837A-Flag). Anti-EGFR and anti-Flag antibodies show expression of EGFR-Flag, and anti-tubulin antibody shows loading control. (D) Doubling time of CHO, CHO-EGFR-Flag and CHO-EGFR-D837A-Flag cells show that CHO-EGFR-Flag grows faster than CHO. (**E**) 0.05T and 1T SMFs reduce cell number in CHO-EGFR-Flag but not the kinase-dead mutant. Relative cell numbers of CHO-EGFR-Flag or CHO-EGFR-D837A-Flag cells after 3 days treatment in 0, 0.05 or 1T SMFs are shown. (**F**) Representative Western blots to examine the downstream components of EGFR in CHO-EGFR-Flag cells. *p < 0.05, **p < 0.01, ns, not significant.

Next we asked whether EGFR is inhibited by SMFs in cells, and whether its kinase activity is critical for cells respond to SMFs, using cell-based assays (Figure S2A) [27, 32]. We compared five different cell lines, including human colon cancer HCT116 cell line, human nasopharyngeal carcinoma CNE-2Z cell line, human cervical cancer HeLa cell line, human retinal pigment epithelial RPE1 cell line and Chinese Hamster Ovary CHO cell line. We used Western Blots to examine the EGFR expression and phosphorylation level and found that EGFR is highly expressed and phosphorylated in HCT116 and CNE-2Z cancer cells but not in CHO cells (Figure 2A). Although CHO cells do not express EGFR, they do have the downstream signaling components. So we chose CHO as a negative control because it provides a null background for EGFR transfection experiments. Our results show that CHO cell proliferation was not affected by 0.05T or 1T SMF (Figure 2B), which is consistent with previous report that demonstrated its insensitivity to even 10-13T strong SMF [33, 34]. We then constructed CHO cell lines that stably expressed wild-type EGFR with a Flag tag (CHO-EGFR-Flag) or kinase-dead mutant (D837A, with no kinase activity) EGFR with a Flag tag (CHO-EGFR-D837A-Flag) (Figure 2C). Wt, but not kinase-dead EGFR caused an increase in proliferation rate in the absence of magnetic field (Figures 2D, S2B). This is consistent with the well-known role of EGFR in cell proliferation. The spontaneous EGFR phosphorylation level in CHO-EGFR-Flag cells was inhibited by 1T SMF (Figure S2C), which indicates that EGFR activity is also inhibited by SMF in cells. In addition, a 1T field caused a reduction in proliferation in cells expressing wt, but not kinase-dead EGFR (Figure 2E), which suggests that the kinase activity inhibition is the major reason for SMF-induced cell growth inhibition in CHO-EGFR-Flag cells. Furthermore, the downstream components of EGFR in CHO-EGFR-Flag cells are also inhibited by SMFs (Figure 2F). Therefore, the data thus far demonstrate that both the autophosphorylation and proliferation-enhancing activities of transfected EGFR can be inhibited by a 1T SMF in living cells.

### STM reveals that SMFs align EGFR kinase domain proteins and interrupt their inter-molecular interactions in solution

**Figure 3:**
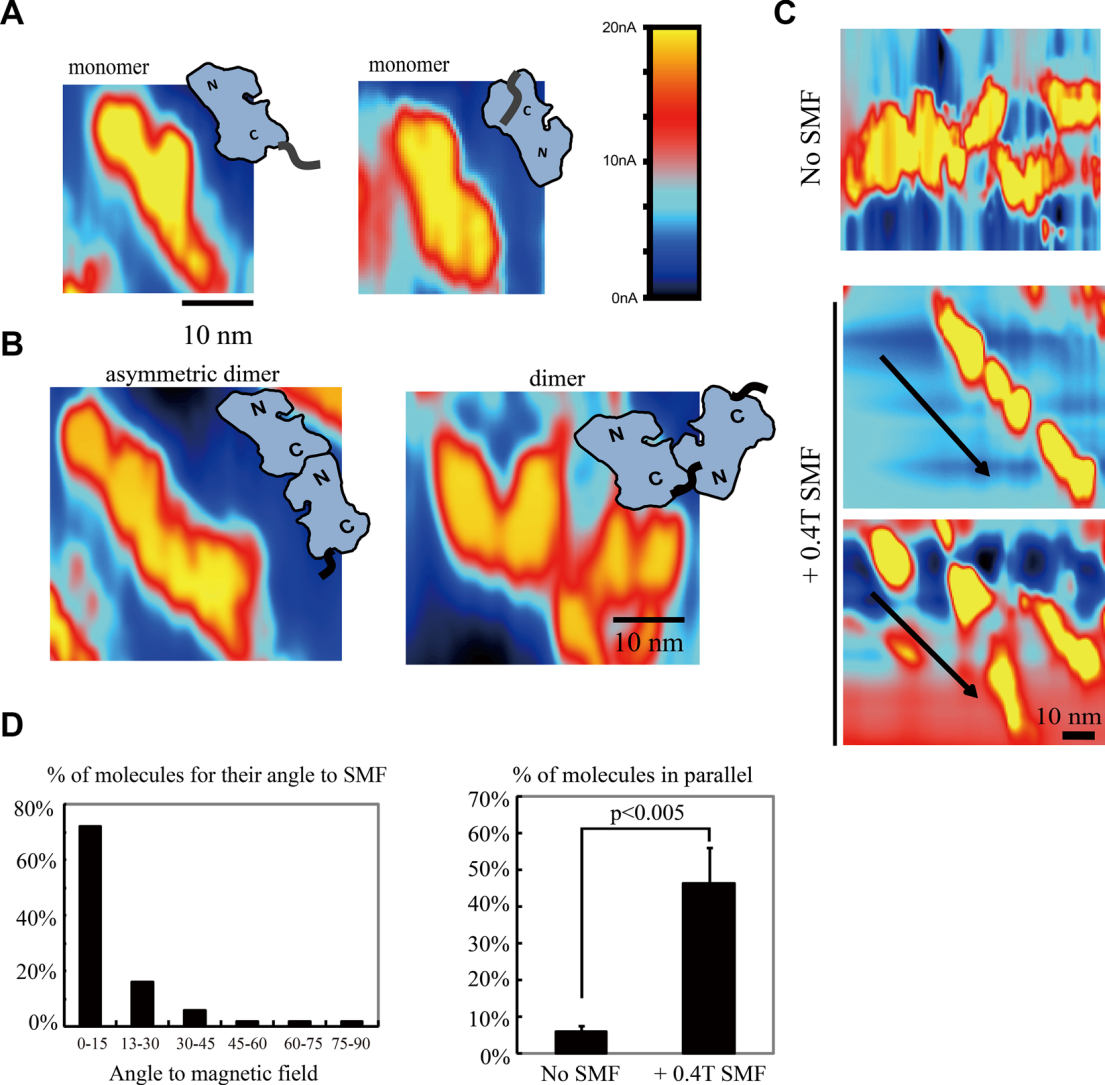
Scanning tunneling microscopy (STM) reveals that SMFs can align EGFR kinase domain proteins in solution (**A**) STM images of EGFR monomers in control condition, with no magnetic field. “N”: N lobe (smaller lobe), “C”: C lobe (larger lobe, connected to its C-terminal tail). Colors represent different tunnel current value, as constant height scanning mode is used in our experiment, it also reflects the ups and downs of the sample. (**B**) STM images of EGFR dimers in control condition, with no magnetic field, in solution. (**C**) A 0.4T SMF aligns EGFR kinase domain proteins. Representative STM images of EGFR kinase domain proteins with or without 0.4T SMF. Arrows show the direction of the magnetic fields. (**D**) Quantification of the angles between EGFR kinase domain protein long axis with the 0.4T magnetic field (left) and percentage of EGFR kinase domain proteins that are in parallel with each other with or without 0.4T SMF (right). Calculation was based on around 150 EGFR molecules from three independent experiments (*n* = 3). Error bars represent SD. Scale bars: 10 nm.

To investigate the molecular mechanism of EGFR inhibition by SMFs, we used Scanning Tunneling Microscopy (STM). Based on the application of STM in electrochemistry studies, we designed an STM with exceptionally low drift, low leakage current, high stability and high precision that enabled us to directly observe the EGFR kinase domain at single molecule level in solution (Figures 3 and S3). We then visualized EGFR with or without a SMF applied from the side. In control conditions without SMF, EGFR proteins were randomly distributed in solution and loosely settled down on the supporting graphite. EGFR kinase domains were frequently visualized as monomers (Figure 3A) and dimers (Figure 3B). The orientation of most EGFR molecules can be distinguished by the relative size of the two lobes (the C terminal lobe of EGFR kinase domain is larger than the N terminal lobe) and by the location of C-terminal tails. Our STM images show that they can form asymmetric dimers in solution, with the bigger C lobe interacts with the smaller N lobe, which is required for their activation (Figure 3B, left panel). However, since the molecules were all in solution and their orientations were not fixed, the C-terminal tails and the detailed structural information were sometimes hard to distinguish (Figure 3B, right panel). But we found that application of a 0.4T SMF significantly changed the protein orientation by causing many EGFR monomers to align along the magnetic field in solution (Figures 3C and S3). To present the STM data in a more quantitative way, we quantified around 150 EGFR protein monomers according to their orientations in solution from three independent experiments. Quantification showed that around half of the proteins were aligned close to the magnetic field direction and in parallel with each other (Figure 3D). We hypothesis that this high alignment effect is partially due to the high local protein concentration (proteins are in close proximity and may have some liquid crystal phase behavior) and the two dimensional restraints in STM measurements (the substrate provides a support for the proteins so that they can only move laterally).

**Figure 4:**
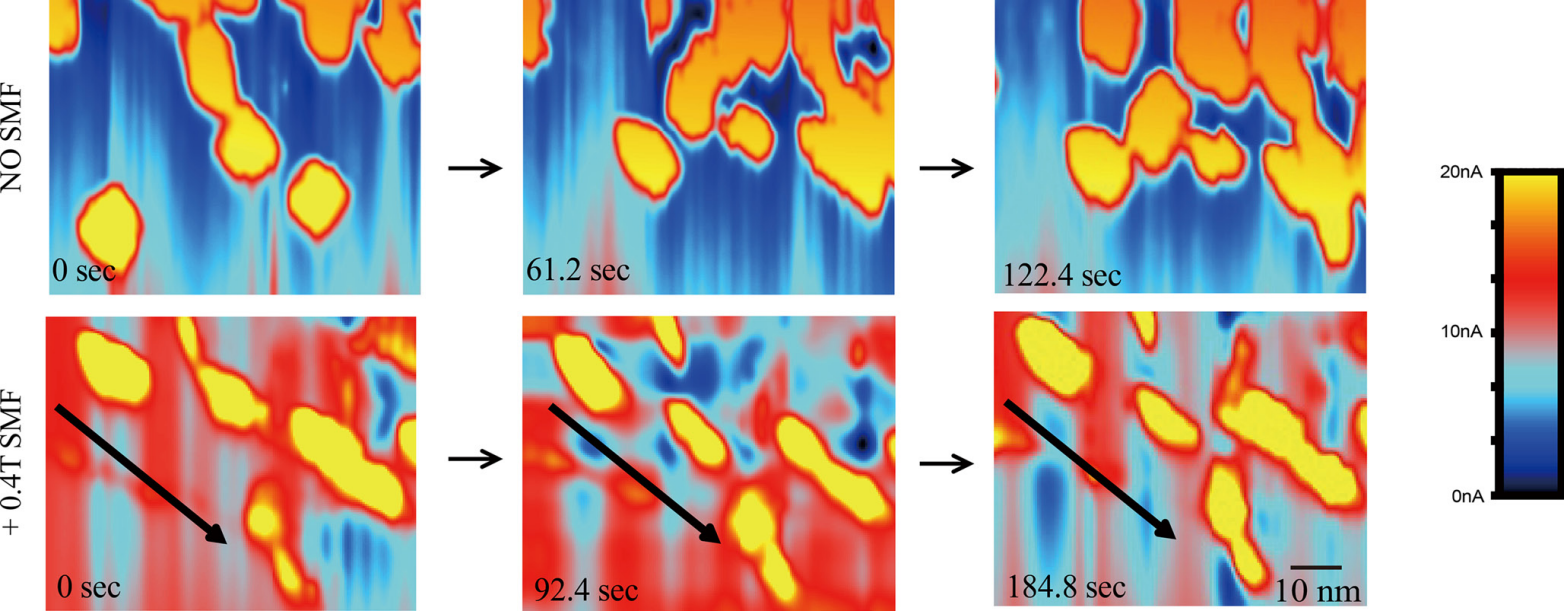
Time-lapse STM reveals that SMFs can align EGFR kinase domain proteins to prevent inter-molecular interactions in solution Time-lapse L-STM images of EGFR with or without 0.4 T SMF. Arrows show the direction of the magnetic fields. Color represents different tunnel current value. Scale bar: 10 nm.

We also took advantage of STM in solution for its ability to visualize these proteins in motion by taking time-lapse STM images. In control conditions, the EGFR kinase domain proteins in solution could collide into each other to form transient dimers/oligomers (Figure 4, upper row), which likely contributes to their basal phosphorylation levels in kinase assays *in vitro*. By imaging during application of the SMF we were able to directly visualize orientation of EGFR domains with the magnetic field (Figure 4, lower row). This alignment effect of SMF was consistent, and likely interrupted interactions between monomers to prevent kinase domains from forming asymmetrical dimers required for trans-autophosphorylation.

### Molecular dynamics simulation indicates that strong SMFs affect orientation of EGFR kinase domain proteins

**Figure 5:**
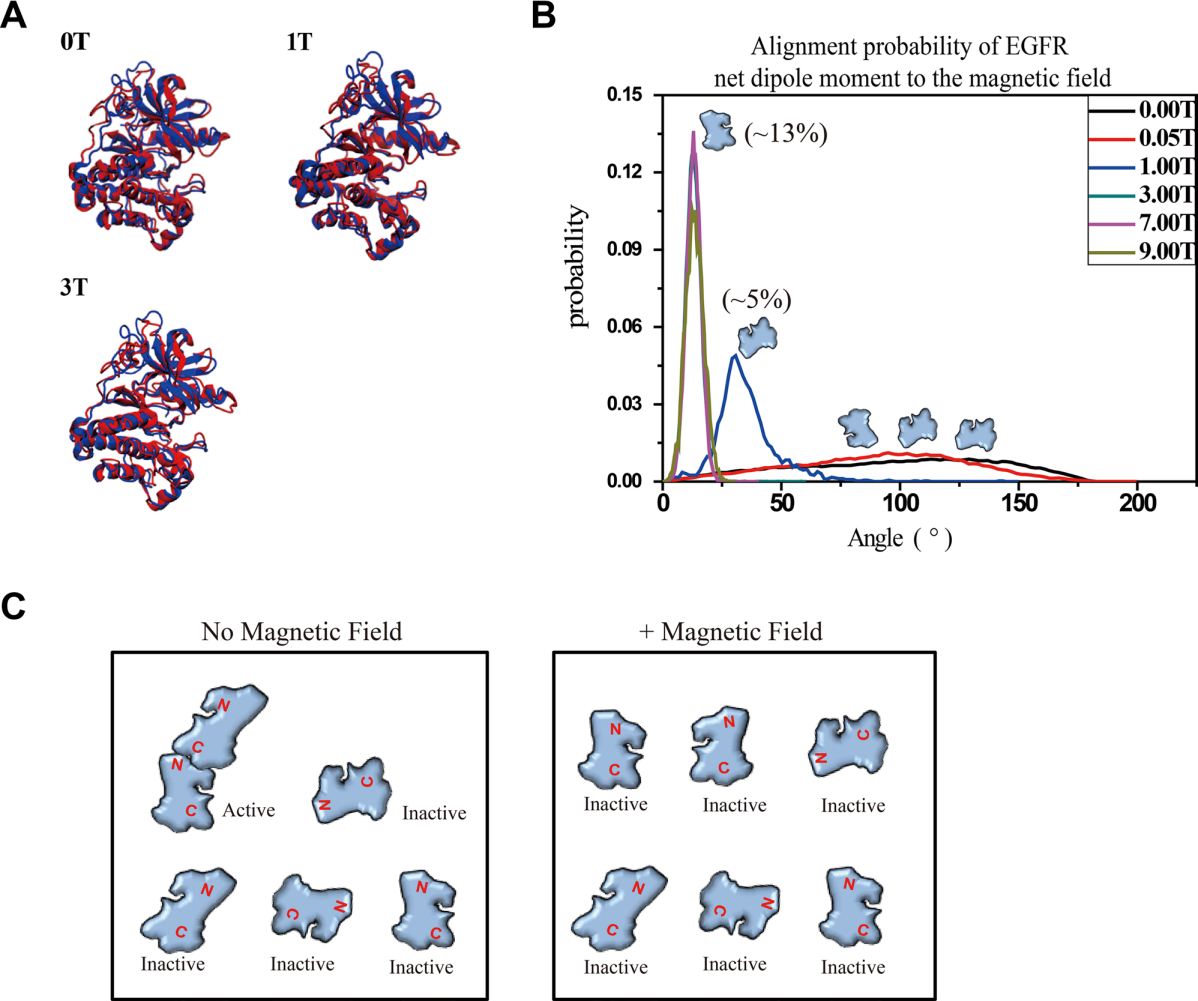
Molecular dynamics simulation shows that 1-9T SMFs affect orientation of EGFR-KD (**A**) The superimposed models between crystal structures (blue) of EGFR-KD and the final snapshot of MD under different SMFs (red). (**B**) Probability distribution of the angles between the net dipole moment of EGFR-KD and field direction. (**C**) Cartoons illustrate the effect of SMF on protein alignment and activation.

It should be noted that the *in vivo* the effect of SMF on biomolecules might be dependent not only on their diamagnetic anisotropy but also on the electric dipole oscillations. Coherent vibrations of living cells were reported and are now assumed to be a signature of life [35–37]. The physical properties, diamagnetic anisotropy and coherent electric polar vibrations of biomolecules are all contributing factors for their responses to magnetic field. To further analyze the physical mechanisms, for instance, exerted torque or the Lorentz force acting on oscillating dipoles in EGFR, we turned to Molecular dynamics (MD) simulation. The effect of the magnetic field was implemented in the simulation by DL_POLY package [38, 39]. Our simulation data showed that although the overall structures of EGFR kinase domain proteins were not much affected (Figure 5A), SMFs had alignment effects on EGFR kinase domain proteins, which likely prevented EGFR proteins from forming asymmetric dimers required for activation (Figure 5B, 5C). The calculated distribution of the angle between the net dipole moment of EGFR kinase domain and magnetic field direction showed that 1-9T SMFs affected the orientation of EGFR kinase domains. In addition, stronger SMFs exhibited stronger effects (Figure 5B). Without SMF, the EGFR molecule orientation is random while 1T SMF is able to slightly (5%) increase the probability of this molecule to tilt to around 30° relative to the direction of the magnetic field. At 9T the calculated probability for each single EGFR molecule to align close to the magnetic field direction is around 13% (Figure 5B). It should be noted that although the chance of multiple EGFR kinase domain molecules to align to the same direction simultaneously is very low, it should be significant enough to affect EGFR function, which has a very high requirement for accurate docking between monomers.

### 9T strong SMFs effectively inhibit cell proliferation in CHO-EGFR and EGFR expressing cancer cells

**Figure 6:**
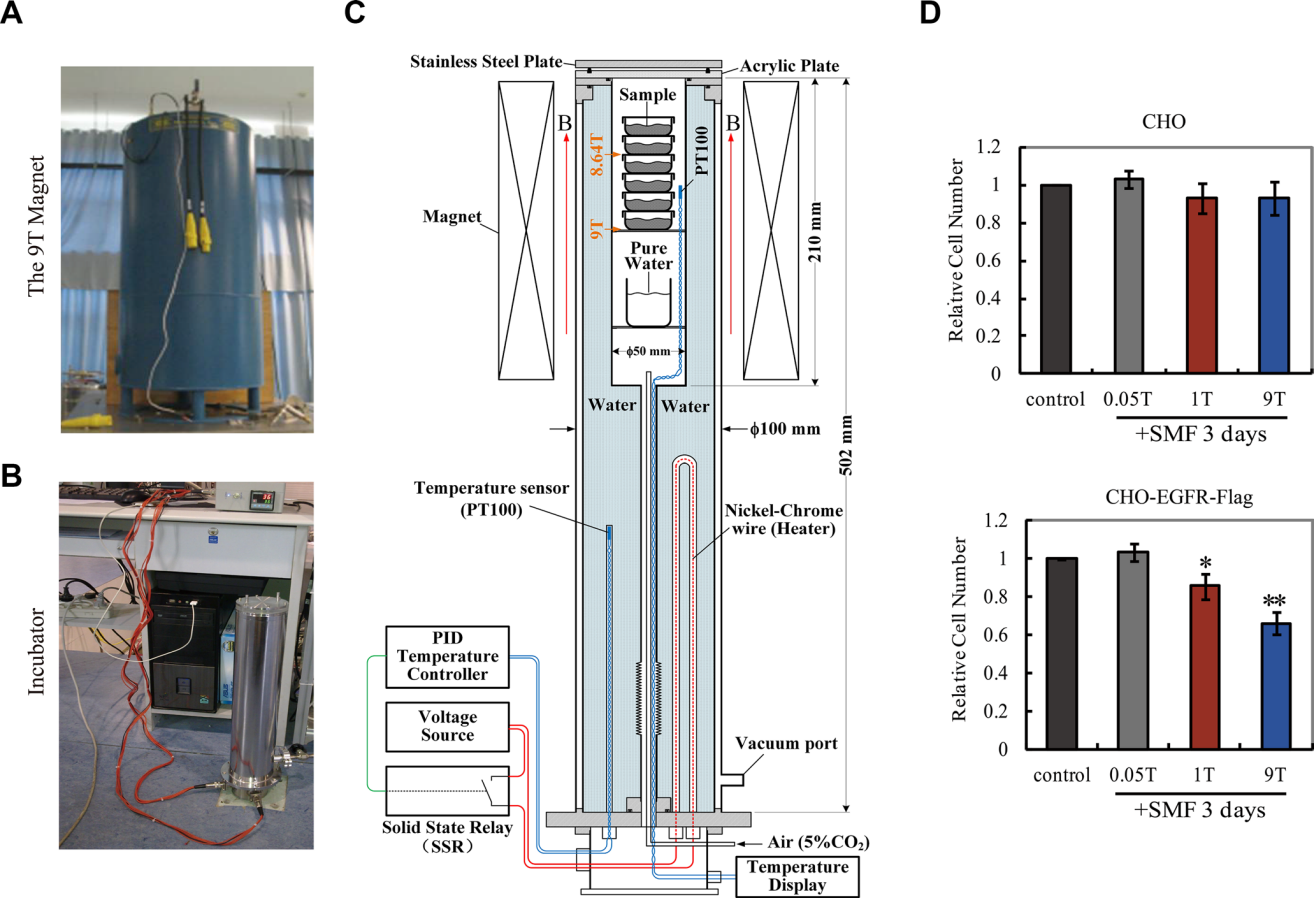
9T strong SMFs have stronger inhibition than 1T SMF on cell proliferation in CHO cells overexpressing EGFR (**A**) The 9T superconducting magnet. (**B**) The cell incubator that fits the 9T magnet. (**C**) The design of the system. (**D**) 9T SMF inhibits CHO-EGFR-Flag cells more efficiently than 1T SMF. Relative cell numbers of CHO and CHO-EGFR-Flag cells after 3 days treatment in 0, 0.05, 1T or 9T SMFs are shown. Quantifications were from three independent experiments (*n* = 3). Error bars represent SD. *p < 0.05, **p < 0.005.

Since our MD simulation data show that stronger SMFs (3-9T) gave stronger alignment effects than 1T SMF (Figure 5B), it is possible that they can also achieve stronger effects in cells. To explore the effects of strong SMFs on EGFR activity and cell proliferation we designed a cell incubator that fits a large bore 9T superconducting magnet (Figure 6A–6C) with accurate controls for temperature, gas and humidity, which enabled cell incubation over multiple days. We first compared the effect of strong fields on control CHO cells vs. CHO-EGFR-Flag expressing cells, as in Figure 2. Control CHO cells were not much affected, confirming the favorable environment for cell growth in the special incubator in the superconducting magnet, while CHO-EGFR-Flag cells exhibited a decrease in proliferation that varied with the strength of the field, and was pronounced at 9T (Figure 6D).

**Figure 7:**
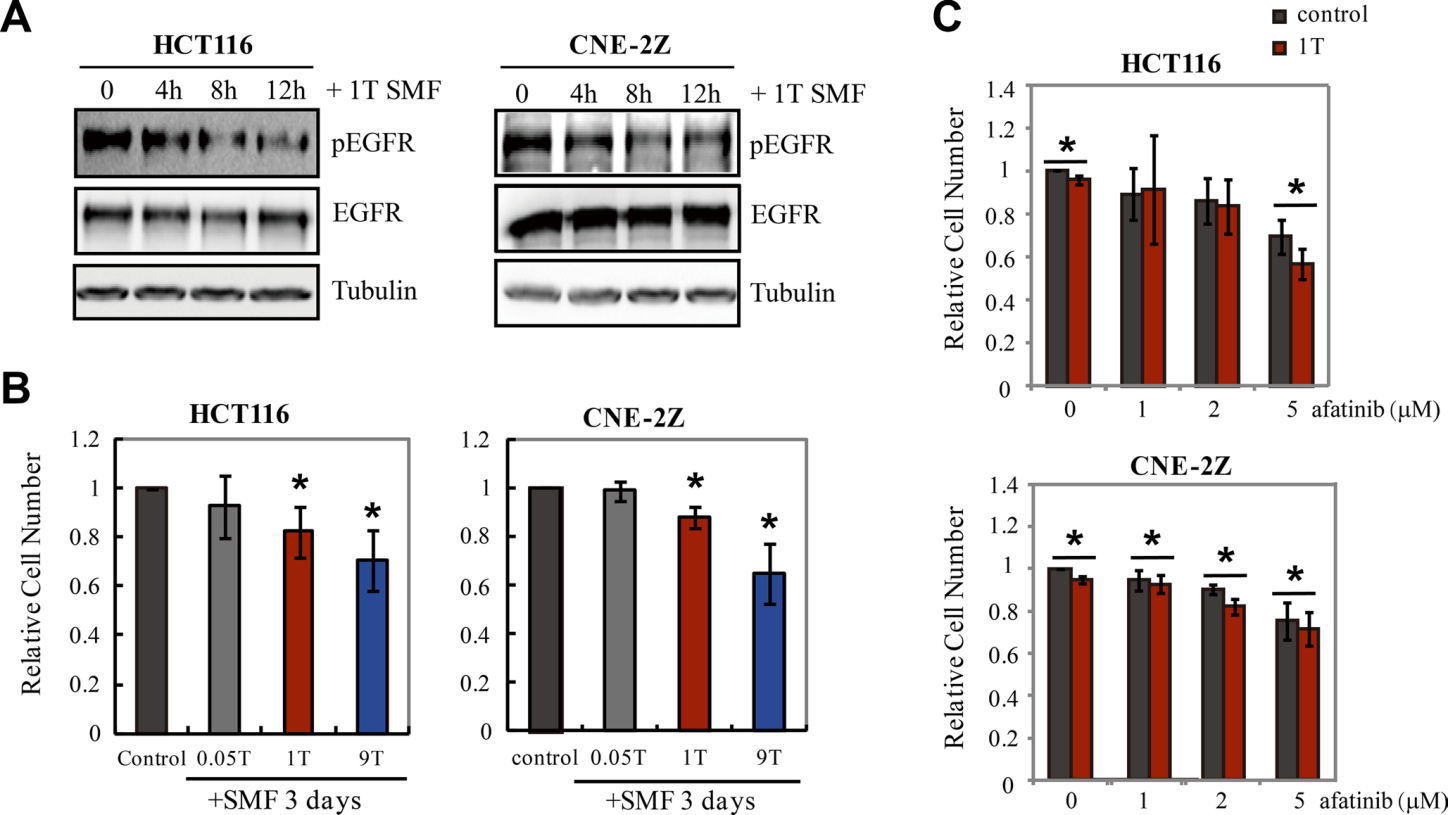
1T and 9T SMFs inhibit EGFR expressing HCT116 and CNE-2Z cancer cells (**A**) EGFR phosphorylation is inhibited by 1T SMF in HCT116 and CNE-2Z cells. Representative Western blots using anti-pEGFR (992) for HCT116 and anti-pEGFR (1068) for CNE-2Z. (**B**) Relative cell numbers of HCT116 and CNE-2Z cells after 3 days treatment in 0, 0.05T, 1T or 9T SMFs. Quantifications were from four independent experiments (*n* = 4). (**C**) Combinational effects of 1T SMF and an EGFR inhibitor afatinib on HCT116 and CNE-2Z cell proliferation. Cells were treated with 1T SMF and afatinib for 3 days. Quantifications were from three independent experiments (*n* = 3). Error bars represent SD. *p < 0.05.

To investigate the therapeutic potential of strong SMFs, we next examined effects on two human cancer cell lines that express EGFR, HCT116 and CNE-2Z. Phosphorylation of EGFR in both cell lines was inhibited by 1T SMF (Figure 7A). In addition, 9T SMF inhibited proliferation to a greater extent than 1T SMF. After 3 days exposure, 1T SMF reduced HCT116 cell density by 18 ± 10 % (*p* < 0.05) and CNE-2E cells by 16 ± 6 % (*p* < 0.05) while the 9T SMF reduced the HCT116 cells by 29 ± 12 % (*p* < 0.05) and CNE-2E cells by 29 ± 13 % (*p* < 0.05) (Figure 7B, Figure S4). The efficiency of the 9T strong SMF to induce cancer cell number reduction is approximately two-fold that of a 1T SMF. In addition, we combined 1T SMF with an EGFR inhibitor afatinib and found that 1T SMF could increase the inhibition effects of afatinib on cell proliferation of both HCT116 and CNE-2Z cells (Figure 7C).

## DISCUSSION

Although we only tested purified EGFR kinase domain protein *in vitro*, we predict that the full-length EGFR is also inhibited by SMFs. The full length EGFR protein is large and has hydrophobic transmembrane parts that complicate the experimental setup so that we used the kinase domain of EGFR in our pure protein studies, which is the key domain responsible for EGFR activity. Our cellular studies show that the endogenous EGFR phosphorylation is inhibited in cells by SMFs. Additionally, the transmembrane and juxtamembrane domains of EGFR, both of which contribute to EGFR activation by forming specific orientations within dimers, are mostly composed of alpha helices [14, 15]. It is known that alpha helix has large diamagnetic anisotropy because the planar peptide bonds are oriented parallel to the helix axis which is the axis of smallest numerical diamagnetism [26]. Therefore, we predict that the orientation of transmembrane and juxtamembrane regions may also be affected by SMF, which inhibits EGFR activation. In addition, SMFs could also affect lipid membrane composition and/or properties, which affect EGFR [40] and contribute to the cellular effects of SMFs on cells. The glycosylation of EGFR and the orientation of EGFR ectodomain relative to cell membrane [41], which are crucial for EGFR activity, may also be affected by SMFs.

An outstanding question is why we do not see this protein alignment in NMR (Nuclear Magnetic Resonance) study. We propose that there are two major reasons for the high alignment effect in our STM study. First of all, the supporting graphite provided a platform for EGFR to settle on, which constrained EGFR proteins to a 2D space, like the cell membrane in living cells. In addition, the measured area often has multiple protein molecules. This mimics the high local concentration of EGFR kinase domains on the cell membrane in cells, which is induced by receptor dimerization. This high local concentration is likely to render them liquid-crystal behavior, which makes them more susceptible to magnetic field. Although the NMR study of the whole EGFR kinase domain has not been done, we predict that the alignment effect in NMR will not be very high. First of all, our MD analysis shows that for each single EGFR kinase domain molecule, there is only around 10% probability that it will align close to the magnetic field direction. In the NMR studies, which measure a large population of proteins, the chance of multiple EGFR proteins to simultaneously align along one direction (the magnetic field direction) will be much lower.

STM is well suited to visualize the dynamics effects of SMFs on proteins. In NMR, a magnetic field is always present, while crystallography and electron microscopy do not visualize dynamics. Also, crystal packing forces may dominate SMF-induced forces in crystallography. However, due to technical limitation with previous instruments, the biological effect of SMF at the single molecule level has never been reported by direct microscopic observation before. Here we designed a homebuilt STM in solution and used it to determine the effects of SMF on the EGFR kinase domain and found that SMF can affect the protein orientation to inhibit their activation. Our study not only demonstrates the new advances of using STM in biological samples in aqueous solution at the single molecule level, but also shows that the intermolecular interaction of EGFR is affected by SMF.

As people already know that the cell is an anisotropic object filled with anisotropic structures, such as mitochondria, microtubules and the cell membrane, an obvious question regarding our finding is how unique EGFR is. As mentioned above, EGFR has a special activation requirement for orientation. Even a slight change in its orientation can obviously affect its activity. This is a very important factor in its magnetic susceptibility. In addition, it has a large number of tyrosines, which contain aromatic rings and have higher diamagnetic anisotropy than regular peptide bond. Here in this study we focus our study on EGFR overexpressing cells (CHO-EGFR cells and the EGFR overexpressing cancer cell lines). In many cancer types, EGFR is the driven force that makes these tumor cells grow faster than normal. Therefore, our study shows that the magnetic field can inhibit EGFR and bring the growth rate back to the normal level. However, it is unlikely that EGFR is the only magnetically responsive structure in cells. Since EGFR is able to form heterodimer with other EGFR family members upon different ligand treatment, we predict that it is very likely that SMF could affect the interaction of EGFR with its family member as well. In addition, we predict that ion channels and other membrane receptors, especially RTKs (Receptor Tyrosine Kinases) are likely to be affected by magnetic field as well. It needs to be investigated specifically about whether and how strong a SMF is needed to affect a given protein. From a practical perspective, it may affect other regulatory proteins and ion channels, and thus broaden the potential applicability of strong magnetic fields in medical environment.

For the magnetic field strength, the 0.4 T and 1 T SMFs used in this study are close to the patient exposure intensity in many hospital MRI scanners. Although high-field MRI scanners are already in clinical and preclinical stages with no obvious adverse effects, International Commission on non-ionizing radiation protection (ICNIRP) currently recommends an exposure limit of human body to 0.4 T (400 mT) [42]. The patient exposure time to MRI in the hospital is usually around a few minutes to 1 hour. While our purified protein assays (kinase assays and STM assays) are around that time range, our cellular studies used longer exposure (4 hours to 3 days) because we wanted to look into cellular responses to prolonged magnetic field treatment. In addition, it has been suggested that moderate intensity SMFs have the potential as an adjuvant treatment method for clinical chemotherapy because they could have combinational effects with some chemotherapy drugs [7, 27, 32, 43, 44]. In fact, we found that 1 T SMF can suppress the mTOR inhibitor-induced EGFR reactivation and enhance the anti-tumor efficacy of mTOR inhibitors [32] and EGFR inhibitor afatinib.

There is growing evidence to show that SMFs can inhibit cancer cell growth but there are still some reservations and uncertainties about these reports. One major concern is the absence of mechanisms that can explain this phenomenon, which is further complicated by the differential biological effects caused by multiple experimental variations. Our results demonstrate that SMFs can directly affect the EGFR kinase domain protein orientation to inhibit its activity in a magnetic field intensity-dependent way, which plays an important role in SMF-induced cancer cell growth inhibition. Further investigation is needed to explore the effect of SMFs on other proteins, especially other RTKs, other types of membrane receptors and channel proteins, as well as potential clinical applications of strong SMFs in treatment of cancer and other human diseases.

## MATERIALS AND METHODS

### Cell culture

HCT116 and CHO cells were cultured in DMEM (#10-017-CV, CORING Life Sciences) supplemented with 10% (vol/vol) fetal bovine serum (FBS; #1101-500, Pufei Biotechnology, Shanghai, China), 1% (vol/vol) penicillin/streptomycin (P/S;Hyclone), 5% CO_2_, 37^°^C. CHO-EGFR-Flag and CHO-EGFR-D837A-Flag cells were maintained similar to CHO cells, but the medium was supplemented 1 μg/ml of puromycin. CNE-2Z cells were cultured in RPMI-1640 (#10-040-CVR, CORING Life Sciences) supplemented with 10% FBS and 1% P/S at 5% CO_2_, 37^°^C.

### *In vitro* kinase assays

*In vitro* EGFR kinase domain (residues 696-1022) phosphorylation assays were performed in 38.5 μL of reaction buffer containing 50 mM Tris-HCl (pH 7.4), 10 mM MgCl_2_, mixed with 1.5 μL EGFR-KD (initial concentration 1.5 μg/μL) and 10 μL ATP (initial concentration 100 μM). Reactions were incubated at 37°C for 10 minutes for the fixed time point assays and 0, 5, 10, 20, 30, 60 minutes for the time course assays.

*In vitro* B-Raf kinase assay with its substrate MEK1 was carried out similarly to EGFR kinase domain assays, but used B-Raf and MEK1 proteins instead, at 1:1 ratio. Reactions were incubated at 37°C for 30 minutes. The *in vitro* kinase assays were terminated by addition of 2 × SDS loading buffer (20 mM Tris-HCl pH8.0, 100 mM DTT, 2% SDS, 20% Glycerol and 0.016% Bromophenol Blue) and heated at 95°C for 5 minutes. The results were tested by Western Blotting. Phosphorylated EGFR at the 992 tyrosine residue was detected using anti-phospho-EGFR-992-tyrosine antibodies (#2235P, Cell Signaling Technology, Boston, USA) and phosphorylated MEK1 were detected using anti-phospho-MEK1/2 antibodies (#9154S) (Cell Signaling Technology, Boston, USA). Experiment was repeated for three times for EGFR and two times for B-Raf. Quantification was done by Image J.

### Cells treated with SMFs

Cells were plated in 35mm plates and treated with magnetic field for the indicated time (as labeled in the figures or figure legends). For shorter time-points assays (within 12 hours), 2–3 × 10^6^ cells were plated for each plate, and then treated after 24 hours. For longer time assays (3 days), 3–4 × 10^5^ cells were plated for each plate. For assays combining 1T SMF with the EGFR inhibitor afatinib, CNE-2Z and HCT116 cells were plated in 24-well plates (1 × 10^5^ cells per well) for one day to allow cells to attach. Then cells were treated with afatinib or afatinib combined with 1T magnetic field for another 3 days before they were trypsinized and counted with hemocytometer.

The 0.005T-1T magnets were from China Dafeng Zhongxin Permanent Magnet Material. To ensure the proper cell culturing conditions, magnets were placed in the 37^°^C CO_2_ cell incubator, and the cell culture plates were placed right on the top of the magnets. For cells treated with 9T SMF, 6 plates in which 3–4 × 10^5^ cells were plated for each plate were placed in 5% CO_2_ at 37^°^C in a special incubator under 9T SMF for 3 days. The cells were then taken out of the magnetic fields and images were taken by DSZ2000 microscope (UOP, Chongqing, China) equipped with ISH300 3.0 MP camera. Experiments were repeated for least three times, and representative images were shown in Figures.

### Scanning tunneling microscopy (STM)

Our homemade STM features an ultra-compact scan head in which a piezoelectric tube scanner of 4.6 mm outer diameter by 8 mm length by 0.5 mm wall thickness is co-axially mounted on one end of a polished sapphire rod of 5 mm diameter by 15 mm length, which is spring-pressed on a pair of compact tantalum rails machined from one piece. The sapphire rod served as the linear motion shaft, which is pushed by a stacked Gecko Drive piezo motor for coarse approach. When the coarse approach is done, the piezo motor withdraws and is completely detached from the scanner, resulting in a minimized mechanic loop between tip and sample for ultra-stable image scanning. Our sample pool is a common O-ring sealed Teflon cell used in EC-STM. The 0.25 mm diameter annealed 90%:10% Pt/Ir wire was from Alfa Aesar. Highly Oriented Pyrolytic Graphite (HOPG) was from SPI. The 0.04 mm diameter annealed platinum wire (Purity: 99.99%, from Goodfellow).100 μl Milli-Q water was added into 20 μl EGFR protein for dilution before injected onto the HOPG substrate. We allowed 30 minutes for the protein molecules to settle down and all imaging process was finished within three hours to prevent potential protein degradation.

### Molecular dynamics simulations

All of the Molecular Dynamics simulations were performed using the DL_POLY-4.0.7 program [38]. The initial heating and pre-equilibration (10 ns) were completed by Gromacs-4.5 molecular dynamic code [45]. The initial conformations of the EGFR were derived from the RCSB Protein Data Bank (code: 2GS6), and were protonated assuming a pH of 7.4 using the H++server (http://biophysics.cs.vt.edu). The protein and ions were described using AMBER03 force-field [46] and the water molecules were described using TIP3P parameters [47]. The temperature was held at 310 K and pressure was maintained at 1 bar using a Nose '−Hoover coupling schemes. The integration step was set to 1fs. Simultaneously, to enhance the statistic sampling, three parallel MD simulations were carried out, final analysis for 10 ns trajectory of each MD simulation was performed using GROMACS tools, VMD [48] and locally written code. The Shake method [49] was used to keep all bonds and angles associating with hydrogen rigid at ideal values. In order to handle long-range electrostatic interactions, the smoothed particle mesh Ewald algorithm was used, with a cutoff length of 12 angstrom. The cutoff length for the Lennard-Jones potential was also set at 12 angstrom.

### 9T strong SMF and the incubator

The vertical superconducting magnet system (SM1) was manufactured by American Magnetics Inc. (AMI). The cryostat has a room-temperature bore of 100 mm diameter. We designed a constant temperature device for cell culture that can fit in the 9T strong SMF. The outer diameter of the device is 100 mm, and the length is 502 mm. The device could work in a 9T superconducting magnet with a 102 mm room temperature bore. A waterproof PT100 and a waterproof Nickel-Chrome wire were used as a temperature sensor and heater respectively. A PID (proportional-integral-derivative) temperature controller, a voltage source, and a solid-state relay were used to control the temperature of the water. The water was thermal isolated from the outside by a vacuum space, which is of benefit to control the water temperature. The temperature of the water can be controlled from room temperature to 100°C, and the precision of the temperature is ± 0.1°C. A sample space was surrounded by the water, and its temperature was controlled by heat conduction. The diameter of the sample space is 50 mm, and the length is 210 mm. A circular acrylic plate was covered on the sample space for thermal isolation because the heat conductivity of the acrylic plate is lower than metal, and the sample space was sealed by a rubber O-ring. A circular stainless steel plate was covered on the acrylic plate, and air space for thermal isolation was sealed by a rubber O-ring. There are two circular multi-hole plates in the sample space. The lower plate was loaded with a cup of pure water for adjusting the humidity of the sample space, and the upper plate was set in the center level of the magnet and loaded with the samples. A PT100 near the samples was used as a temperature sensor and connected to a temperature display to monitor the temperature of the samples. For introducing gas into the sample space to adjust the atmosphere, a thin tube was inserted to the sample space from the bottom of the device.

The superconducting magnet SM1 was designed to have high homogeneity. The Maximum magnetic field strength is at the magnet center (the bottom plate of the six cell culture plates). The homogeneity over 50 mm diameter in horizontal plane and 100 mm along vertical cylinder is within 4%. Our cell culture plate is 35 mm in diameter and 12.5 mm in height. The magnetic intensities of the cell culture plates are within the range of 8-9T. We have tested the cells at six different locations within the incubator and there was no noticeable difference between them.

### Wild type and mutant CHO-EGFR-Flag stable cell lines

The cDNA sequence encoding human EGFR was cloned into pMSCV-puro vector with 3 × Flag tag fused at the C-terminal end. The mutant EGFR-Flag was constructed by site-directed mutagenesis at residue 837 by replacing Asp with Ala. CHO-EGFR-Flag (wild type and D837A) cells were established by the retrovirus system. Retroviruses were packaged by transfecting the plasmid containing EGFR-Flag and two helper plasmids into 293T cells using Fugene 6 (Promega), and the supernatant was harvested after 48 hours. Stable cell lines were established by infection of CHO-K1 cells using virus and selected by 10 μg/ml puromycin. The stable cell lines were maintained in medium containing 1 μg/ml puromycin.

### Statistical analysis

For quantifications throughout the paper, mean values and Standard Deviations are shown. Comparisons between treatments were analyzed by a two-tailed Student *t* test. *P* values are labeled in the Figures for where data were compared.

## SUPPLEMENTARY MATERIALS


