# Supplementary material for: Moderate and strong static magnetic fields directly affect EGFR kinase domain orientation to inhibit cancer cell proliferation
Source: Oncotarget. 2016 May 19;7(27):41527–39. doi: 10.18632/oncotarget.9479 (PMC5173076; doi:10.18632/oncotarget.9479)
Supplement: Supplementary file 1 [file oncotarget-07-41527-s001.pdf]

Moderate and strong static magnetic fields directly affect EGFR kinase domain orientation to inhibit cancer cell proliferation

Supplementary Materials

SUPPLEMENTARY METHODS

Protein expression and purification

A construct encoding EGFR residues 696-1022 with a GST tag was cloned into baculovirus expression vector pAcG2T. The protein was expressed by infecting SF9 cells with high titer viral stocks for 48 h. Cells were harvested and lysed in 25 mM Tris (pH 7.9), 150 mM NaCl, and 1 mM DTT. The supernatant was incubated with glutathione Sepharose beads (Genscript). After washing with wash buffer (40 mM Tris pH 7.9, 500 mM NaCl, 1% Glycerol, 1 mM DTT), the beads were incubated overnight with 5 ml wash buffer containing 5 µl of 5 mg/ml alpha-thrombin to remove GST tag. The eluted EGFR protein was loaded on a desalting column PD-10 (GE) to change the buffer to 25 mM Tris pH 7.5, 50 mM NaCl, 20 mM $MgCl_2$, and 1 mM DTT. The protein was concentrated, flash frozen in liquid N_2, aliquoted and stored in −80°C. Protein concentration was determined by Bradford.

For B-Raf protein, the cDNA fragment encoding the kinase domain of B-Raf was cloned into a pTriEx vector (Novagen), and B-Raf protein was expressed by transfecting the plasmids into 293T cells using Fugene 6 (Roche). After 48 hours, 293T cells were collected and lysed in 50 mM Tris, pH 7.4, 100 mM NaCl and 2 mM EDTA, 1 mM PMSF and 1% NP40. The supernatant was incubated with Ni-resin (Qiagen). The beads were then washed by lysis buffer containing 50-250 mM imidazole. The elute was loaded to PD-10 desalt column and changed into 50 mM Tris pH7.4 150 mM NaCl 20 mM $MgCl_2$ and 1 mM DTT. The protein was concentrated to 1 mg/ml and aliquots were frozen and stored at −80°C.

For MEK1 protein, the cDNA fragment encoding the full length of MEK1 was cloned into pet22b vector. The plasmid was transformed into E. coli BL21 (DE3) plysS strain and protein expression was induced by addition of 1 mM IPTG when cell density reached 0.6 OD600 units. Cells were harvested and re-suspended in lysis buffer (25 mM Tris pH 8.0, 150 mM NaCl, and 1 mM PMSF). The cells were lysed by ultra sonication and the cell debris was removed by ultracentrifugation. The supernatant was incubated with Ni-affinity beads (GE). The beads were then washed by lysis buffer containing 50–250 mM imidazole. The elute was loaded to sephedex 75. The protein was concentrated to 1 mg/ml and aliquots were frozen and stored at −80°C.

Cell growth curve and doubling time calculation

1×10^5 cells were plated on 35 mm plates and cell number was counted again 6–8 hours after seeding, when the cells are all attached to the plates. For each cell line, 10 photos were taken using a DSZ2000 microscope under a 10× objective lens every 24 hours. Cell number was counted using Adobe Photoshop CS3 Extended software. Total of around 2×10^4 cells were counted for each cell line. Cell number was normalized to day 1 and relative cell numbers are used to generate growth curve for each cell line. Cell growth doubling time was calculated using formula: $Td = t*\log_2/(\log N_t – \log N_0)$. "t" is incubation time; "N_0" is first counting cell number; "N_t" is cell number after incubation time. In this experiment, "N_0" and "N_t" are both relative cell number. Data show mean ± s.d. for two independent experiments.

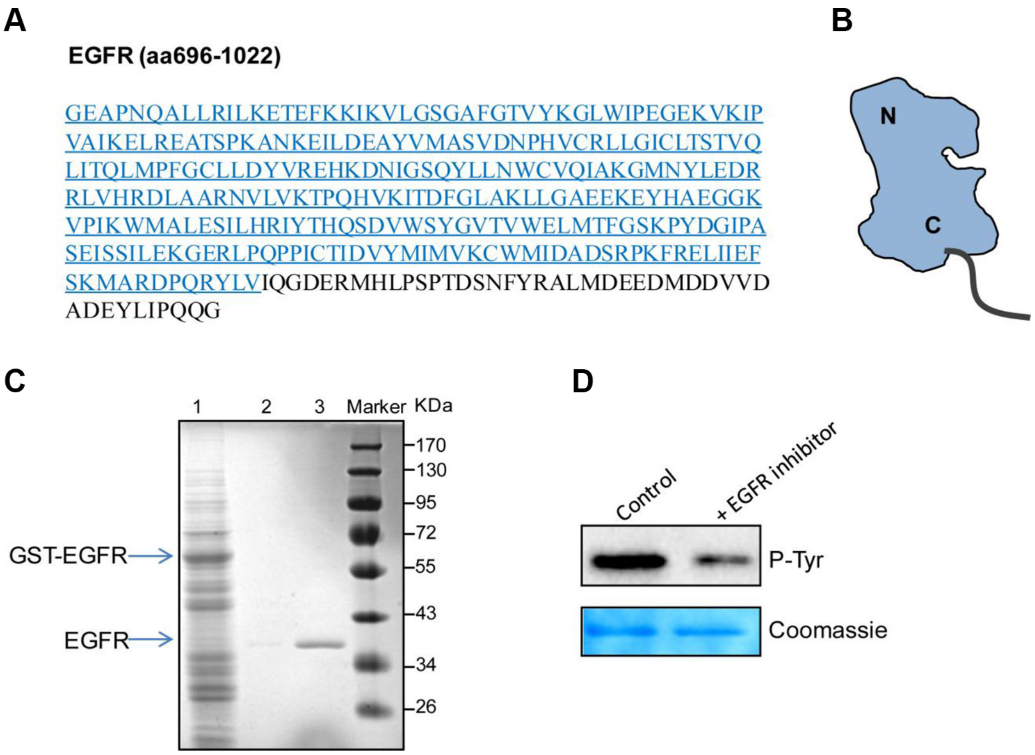

Supplementary Figure S1: EGFR kinase domain sequences and its kinase activity. (**A**) Sequence of the EGFR kinase domain protein (EGFR-KD, aa696-1022) used in this study, which is composed of the core kinase domain (blue font, underlined) and a short C terminal tail. (**B**) A model illustrates the conformational information of the EGFR kinase domain protein with the C terminal tail. (**C**) EGFR-KD purification. 1. Cell lysate expressing GST (Glutathione S-transferase) tagged-EGFR-KD. 2. Purified protein after thrombin digestion, which cut off the GST tag. 3. Purified and concentrated EGFR-KD protein. (**D**) EGFR kinase assays in the absence or presence of the EGFR inhibitor pelitinib (5 μM). Upper images show the Western blot analysis using an anti-phospho-tyrosine antibody. Lower image shows the coomassie blue stain.

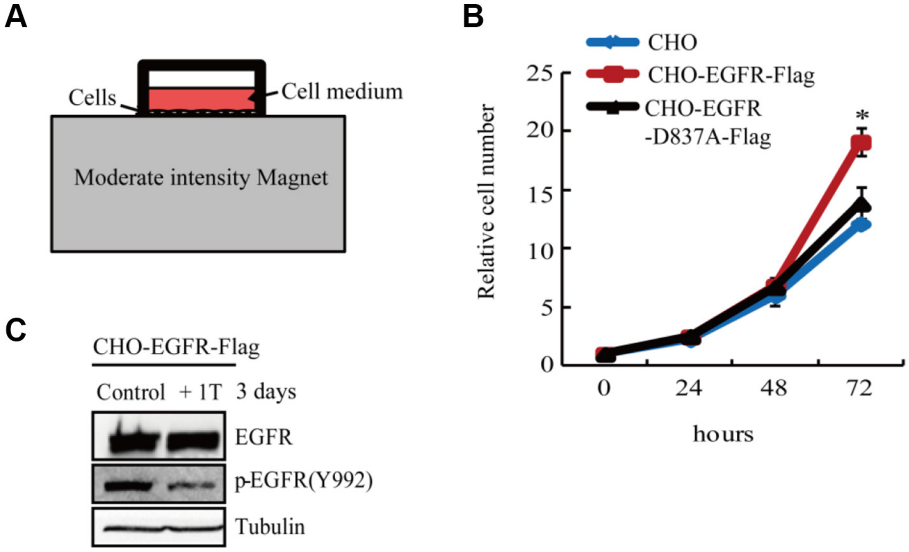

Supplementary Figure S2: EGFR overexpression promotes cell proliferation but its phosphorylation is inhibited by 1T SMF. (**A**) Illustration of the cell culture plate on the surface center of a magnet. Red part shows cell culture medium and the cells are in the bottom. (**B**) Growth curve of CHO, CHO-EGFR-Flag and CHO-EGFR-D837A-Flag cells show that CHO-EGFR-Flag grows faster than CHO. (**C**) 1T SMF inhibits EGFR phosphorylation in CHO cells stably expressing EGFR-Flag. Representative Western blots of CHO-EGFR-Flag cells with or without 1T SMF treatment for 3 days. Anti-pEGFR (Y992) shows the phosphorylation level of EGFR, and anti-EGFR was used to show total EGFR protein.

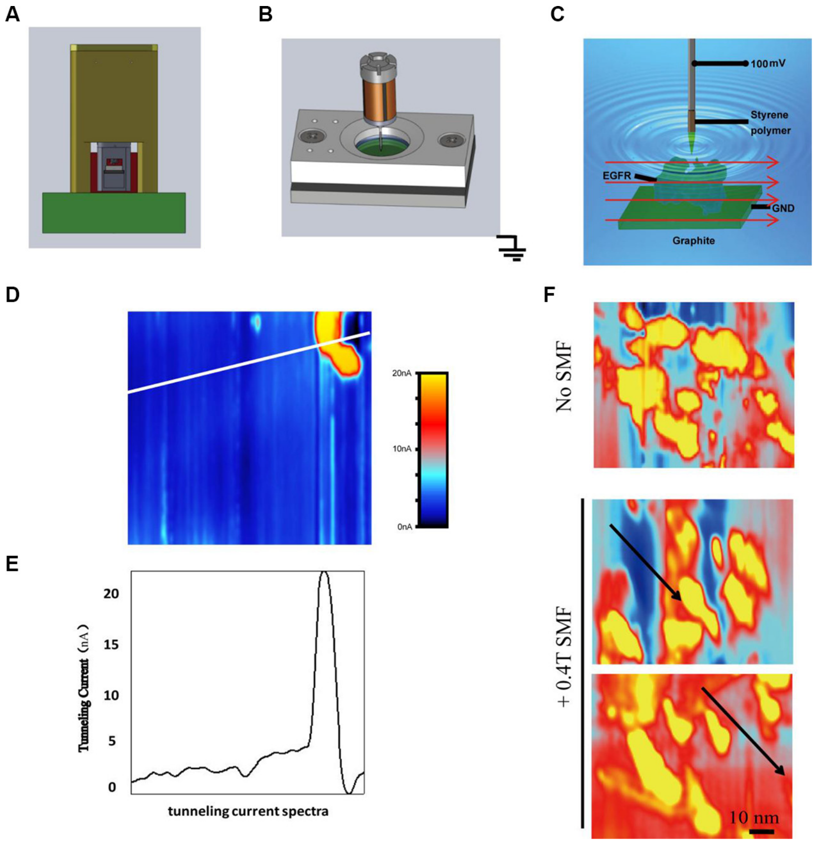

Supplementary Figure S3: Using STM to observe EGFR kinase domain protein in solution. (**A–C**) Illustration of the experimental set-up for the scanning tip, EGFR kinase domain protein and the substrate. Red arrows show the direction of the magnetic fields. (**D**) A STM image of an EGFR kinase domain protein in solution. The white line shows the cross-section that was measured. Colors represent different tunnel current value, as constant height scanning mode is used in our experiment, it also reflects the ups and downs of the sample. (**E**) Cross-sectional tunneling current profile for (D). (**F**) A 0.4T SMF aligns EGFR kinase domain proteins. Representative STM images of EGFR kinase domain proteins with or without 0.4 T SMF. Arrows show the direction of the magnetic fields. Scale bar, 10 nm.

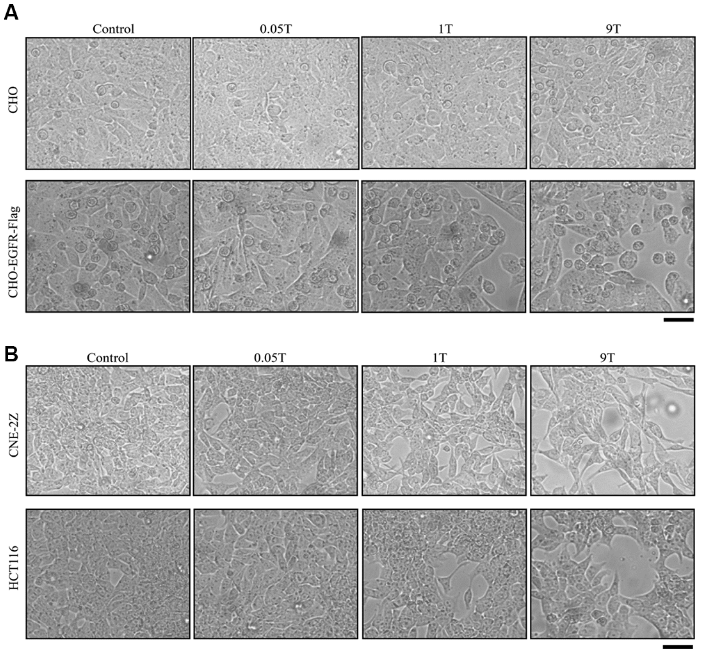

Supplementary Figure S4: 1T and 9T SMFs inhibit CHO-EGFR-Flag cells as well as HCT116 and CNE-2Z cancer cells. Representative bright field images of (**A**) CHO and CHO-EGFR-Flag cells, or (**B**) HCT116 and CNE-2Z cells treated with 3 days of 0, 0.05, 1 or 9T SMFs. Scale bars, 20 µm.
